# Editorial: Molecular architecture and dynamics of meiotic chromosomes

**DOI:** 10.3389/fcell.2024.1386038

**Published:** 2024-03-04

**Authors:** Ricardo Benavente, Mónica Pradillo, Pedro A. San-Segundo

**Affiliations:** ^1^ Department of Cell and Developmental Biology, Biocenter, University of Würzburg, Würzburg, Germany; ^2^ Department of Molecular Biology, Instituto de Investigaciones Biológicas Clemente Estable, Montevideo, Uruguay; ^3^ Department of Genetics, Physiology and Microbiology, Faculty of Biological Sciences, Universidad Complutense de Madrid, Madrid, Spain; ^4^ Instituto de Biología Funcional y Genómica (IBFG), Consejo Superior de Investigaciones Científicas-Universidad de Salamanca (CSIC-USAL), Salamanca, Spain

**Keywords:** meiosis, chromosome, pairing, synapsis, meiotic recombination, checkpoints, nuclear envelope

## Introduction

Meiosis is a special type of cell division that allows the generation of haploid gametes and is a key process for sexual reproduction of animals, plants and fungi. Haploidization requires that meiotic cells undergo a series of unique processes; namely, pairing, synapsis, recombination and segregation of homologous chromosomes. This involves profound meiosis-specific changes in the protein composition and architecture of homologous chromosomes as well as of the condensation and folding of chromatin that require a critical timing and regulation. The details of these changes may vary among different species. Nevertheless, the essential nature of meiosis has remained highly conserved throughout evolution.

A major goal of the present Research Topic of *Frontiers in Cell and Developmental Biology* is to provide an overview of how meiotic chromosomes and their components are critically involved in the mechanisms of haploidization and how dynamic protein complexes yield important structural intermediates and temporal regulation to this process. To this end, this special Topic contains selected original research and review articles dealing with the composition, architecture, function and regulation of meiotic chromosomes of animals, plants and fungi using microscopic, biochemical, molecular and/or genetic techniques.

This Research Topic comprises 15 articles covering different aspects of Meiosis. For clarity, we have divided them into four main themes: Architecture and recombination, Pairing and chromosome dynamics, Regulation of meiotic progression, and Nuclear envelope functions.

## Architecture and recombination

In recent years, substantial progress has been achieved demonstrating the outstanding role of the chromosome axis in meiosis-specific processes, i.e., pairing, synapsis and recombination (Zickler and Kleckner, 2023). In their article, Ito and Shinohara provide an up-to-date overview on the peculiarities and roles of meiotic axial structures and their role in double-strand break (DSB) generation and regulation, as well as in crossover (CO) formation.

The mini review by Rafiei and Ronceret deals with a still intriguing aspect of meiosis, namely, CO interference (Zickler and Kleckner, 2023). Although this phenomenon has been known for decades, the mechanisms involved have remained elusive. In their article, the authors summarize the data of the literature, particularly in plants, and propose an integrative model for CO interference regulation that involves the synaptonemal complex (SC) as a structure that would allow the diffusion of a CO limiting factor. For their part, Shinohara and Shinohara have also investigated the mechanisms of CO control in budding yeast using cytological and genetic tools. They have explored the relationship between DSB frequencies and the localization of the Msh5 complex in selected strains and concluded that the complex would play an important role in CO homeostasis.

Localization and number of COs are important parameters in meiotic recombination studies. However, the quantitative manual analysis of these events is time consuming. To overcome this limitation, Soriano et al. have developed an ImageJ macro routine that allows for a faster, reproducible, and more rigorous investigation of the mentioned CO parameters in meiotic chromosome spreads of vertebrates. This tool will greatly facilitate the analysis of meiotic COs in the context of the SC, even considering overlapping chromosomes.

The SC is a meiosis-specific nuclear structure that mediates synapsis between homologous chromosomes (Page and Hawley, 2004). Very little is known about the assembly/disassembly process at the molecular level. Pollard et al. have investigated aspects of this process by live-cell imaging in budding yeast using a GFP-tagged Zip1 protein, and they have obtained highly interesting new data on SC kinetics. Notably, while SC assembly occurs with both monophasic and biphasic kinetics, final disassembly takes place rapidly due to Zip1 degradation. In addition, the authors describe a novel type of event, termed “abortive disassembly”, that differs from the final disassembly in various mechanistical aspects.

## Pairing and chromosome dynamics

Traditionally, meiosis has been studied in only a few model organisms. However, the emergence of new tools is enabling researchers to expand the set of model species to include less studied and more unusual systems (Grusz et al., 2017). Studies in these species are really promising and could help answer long-standing questions and provide insight into different strategies for solving meiotic problems. In this context, Marín-Gual et al. analyzed meiotic progression in four reptile species (the Australian central bearded dragon, two geckos and the painted turtle) and demonstrated that the bouquet is a highly conserved structure during prophase I, whereas the level of DSBs is highly variable among vertebrates. Curiously, these reptile species exhibit low recombination rates, and this feature is shared with the American marsupials *Thylamys elegans* and *Dromiciops gliorides*, where RPA and RAD51 foci show an extreme polarization towards chromosome ends, as it has been reported by Valero-Regalón et al. However, the distribution of meiotic DSBs seems to be different in the Australian marsupial *Macropus eugenii*, where DSB markers are present along the entire length of the chromosomes. In addition, bouquet polarization is incomplete and more transient in this species.

The ability of homologous chromosomes to pair is one of the most enigmatic processes that takes place during meiosis. Although there has been some progress in the understanding of this mechanism, it is still a long way from being fully understood. It seems clear that although meiotic recombination is essential to ensure recognition of homologues, there are interhomologous interactions that are not dependent on DSB formation in many organisms (Page and Hawley, 2004; Da Ines et al., 2014; Zickler and Kleckner, 2023). Solé et al. provide an overview of these recombination-independent events that involve different strategies based on chromosome clustering and movement, chromosome structures, proteins and even non-coding RNAs in five model species. Pairing of homologous chromosomes can be disrupted because of the presence of unequal sets of chromosomes, as is the case in organisms with chromosome rearrangements such as Robertsonian (Rb) translocations (Wallace et al., 2002). Ayarza et al. performed a detailed analysis of the inheritance of Rb (metacentric) chromosomes in the offspring of heterozygous males and females for eight Rb chromosomes. Their results show that the number of inherited Rb chromosomes is not a random process. In addition, they found no evidence for a preferential segregation of translocated chromosomes, i.e., segregation bias or meiotic drive.

## Regulation of meiotic progression

The concept of cell-cycle checkpoints was originally introduced by Hartwell and Weinert more than 3 decades ago to define the control mechanisms enforcing the dependency in the order of cell cycle events. According with this notion, checkpoint pathways prevent the initiation of a late event if a previous one has not been successfully completed (Hartwell and Weinert, 1989). Meiosis involves tightly regulated processes such as homologous chromosome pairing, synapsis, recombination, and segregation. The precise coordination between these meiotic events and the progression of meiotic development is essential to ensure faithful distribution of the chromosomes to the gametes (Subramanian and Hochwagen, 2014). A review by Huang and Roig in this Research Topic focuses on the surveillance mechanisms, or checkpoints, monitoring pairing, synapsis and recombination during meiosis in mice. The authors discuss how studies in mouse models provide insights into genetic regulations and the link between meiotic errors and mammalian infertility, offering potential diagnostic value for human infertility.

Protein phosphorylation, resulting from the balance between the action of kinases and phosphatases, plays a paramount role in the regulatory pathways coordinating timely meiotic progression (Kar and Hochwagen, 2021). Among the numerous kinases acting in meiotic cells, cyclin-dependent kinases (CDKs) and polo-like kinases (PLKs) possess a prominent relevance (Tsubouchi et al., 2018). In this Research Topic, Palacios-Blanco and Martín-Castellanos review the crucial role of cyclins and CDKs in orchestrating meiosis-specific events, including the establishment of unique chromosome architecture, homologous recombination, and synapsis. The authors highlight the evolutionary conservation of meiosis-specific cyclins and CDKs, and their diverse functions. They also emphasize the significance of these regulators in guaranteeing the precise transmission of genetic information. In addition, an original research article by Gómez et al. reports two roles for the polo-like kinase PLK1 during mammalian male meiosis, in particular the disassembly of SYCP3 and HORMAD1 from the lateral elements of the SC, and the assembly of the inner centromere at meiosis I. Their results underscore the importance of PLK1 as a master regulator of meiotic progression in mice spermatocytes.

## Nuclear envelope functions

The nuclear envelope (NE) and its associated structures play critical mechanical and regulatory roles during meiosis. Rapid chromosome movements during meiotic prophase I are promoted by the evolutionarily conserved LINC complex composed by SUN and KASH proteins. The LINC traverses the NE connecting the telomeres (inside the nucleus) with the cytoskeleton (outside the nucleus) providing the physical forces for telomere-driven chromosome motion. These movements are critical for proper interhomologous interactions (Burke, 2018; da Cruz et al., 2020; Zetka et al., 2020). Another type of highly organized assemblies embedded in the NE are the nuclear pore complexes (NPCs). Various meiotic functions for the NPCs are beginning to emerge. In yeast, basket nucleoporins appear to mediate interactions of meiotic chromosomes with the NE (Komachi and Burgess, 2022), and these nucleoporins undergo a dynamic reorganization during meiotic divisions (King et al., 2023). NPCs also contribute to control of meiotic progression by regulation of SUMOylation (Yang et al., 2023). In addition, exportin-dependent nucleocytoplasmic trafficking via NPCs also plays important meiotic roles in yeast and mammals (Onuma et al., 2018; Herruzo et al., 2023). Three articles in this Research Topic address NE-related subjects.

A research article by Gurusaran et al. reports the crystal structure of the luminal coiled-coil domain (α1) of SUN1, which forms a parallel trimeric structure. The trimer is stabilized by zinc coordination via a central cysteine motif. The α1 domain combines with another coiled-coil domain (α2) to mutually reinforce SUN1 trimerization and sustain the interaction with KASH5. This study expands our knowledge about LINC organization and how forces are transduced across the NE to move chromosomes.

In a perspective article, Fernández-Álvarez discusses recent progress in understanding the non-canonical functions of the telomere bouquet during meiosis. High-resolution live-cell imaging techniques combined with data-mining algorithms tracking telomeres, together with advanced quantitative biology, are revealing novel complex chromosome movement patterns and structural features of chromatin. These approaches unveil the plasticity of the telomeric bouquet with higher spatial and temporal resolution.

A research article by Fernández-Jiménez et al. examined the meiotic role of nucleoporins SAR1 and SAR3, which are components of the NPC outer ring in *Arabidopsis thaliana*. Mutation of SAR1 or SAR3 results in abnormal chromatin condensation and chromosomal fragmentation in a subset of meiocytes. These defects are dependent on the formation of SPO11-induced DSBs, and they are also observed in other mutants deficient in the outer ring complex, like *hos1*. Distribution of NPCs is altered in *sar1* mutants. This research provides new insights into how NPCs contribute to meiotic chromosome behavior in plants.

## Concluding remarks

Although our knowledge of meiosis and its biological functions has expanded in recent years, many facets still remain opaque. Comprehensive study of meiosis using innovative techniques will help to elucidate aspects that remain unclear in key meiotic events; namely, pairing, synapsis, and recombination. A deeper understanding of chromosome behavior, including dynamics, movement, and segregation, could also benefit from continued methodological advances. Future studies will allow not just to expand our fundamental scientific understanding, but also to provide valuable practical contributions to several areas including agriculture and healthcare.
